# Assessment the functioning and disability in children with mental disorders

**DOI:** 10.1192/j.eurpsy.2022.581

**Published:** 2022-09-01

**Authors:** L. Díaz-Castro, H. Cabello-Rangel

**Affiliations:** 1 National Institute of Psychiatry Ramon de la Fuente Muñiz, Direction Of Epidemiological And Psychosocial Research, Mexico City, Mexico; 2 Psychiatric Hospital Fray Bernardino Álvarez, Research Division, Mexico City, Mexico

**Keywords:** Mental Disorders, functioning, WHODAS 2.0, Children

## Abstract

**Introduction:**

Despite youth’s high Global Burden of Disease there is a substantial service delivery gap between this population’s urgent needs and their access to health services. Because attention has remained under-prioritized (Babatunde et al., 2019), youth typically do not receive the treatment they require, i.e., they present an unmet need (Barwick et al., 2013). This is particularly problematic given that untreated mental disorders (MD) are associated with short-term and long-term functional deterioration.

**Objectives:**

To determine the level of functioning of children who receive mental healthcare in the selected psychiatric hospitals of Mexico.

**Methods:**

A cross-sectional study was conducted during 2018-2020. Sample of children who received mental healthcare at the time of the study. Questionnaire for the evaluation of disability WHODAS 2.0 (World Health Organization-Disability Assessment Schedule) was applied. T test and analysis of variance were applied to know the differences of means of the variables and indicators.

**Results:**

Sample (n= 397), 63% were boys. Mean (SD) for Age: 12 (3.6) and schooling: 5.8 (3.6). 51% (n =202) of children reported having a generic diagnosis for hyperkinetic disorders and 34% depressive disorder. WHODAS scores: significant differences in the functioning domains (Do). Mean and (SD) for Do5 Life activities domestic: 45 (26.7); Do6 Social participation:37 (20.6); and Do1 cognition: 36.6 (19.3). Figure 1.

**Figure:**
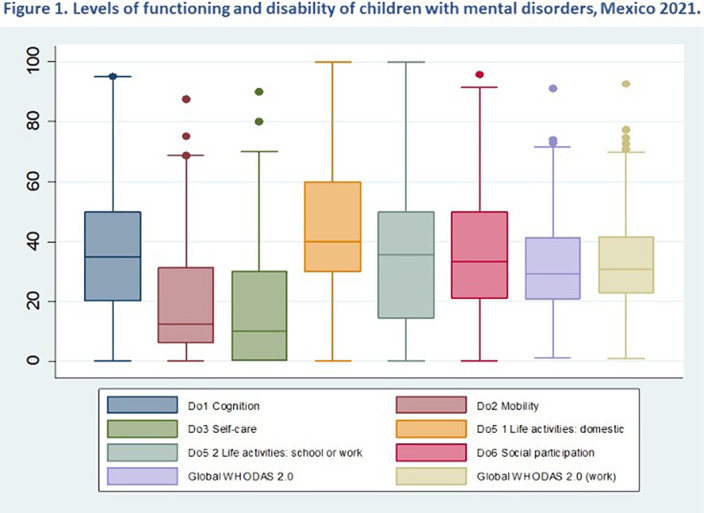

**Conclusions:**

The children with MD are more vulnerable due to the associated disability and it requires specific heath interventions adapted to their mental health care needs. References: 1) Babatunde et al. (2021). Glob.Soc.Welfare 8, 29–46. 2) Barwick et al. (2013). J.evid.based.soc.work, 10(4), 338–352.

**Disclosure:**

No significant relationships.

